# *Klebsiella pneumoniae* Carbapenemase–2, Buenos Aires, Argentina

**DOI:** 10.3201/eid1407.070826

**Published:** 2008-07

**Authors:** Fernando G. Pasteran, Luis Otaegui, Leonor Guerriero, Gabriel Radice, Ricardo Maggiora, Melina Rapoport, Diego Faccone, Ana Di Martino, Marcelo Galas

**Affiliations:** * Instituto Nacional de Enfermedades Infecciosas–ANLIS “Dr. Carlos G. Malbran,” Ciudad Autónoma de Buenos Aires, Argentina; † Sanatorio Trinidad Mitre, Ciudad Autónoma de Buenos Aires

**Keywords:** Carbapenemase, KPC, Klebsiella pneumoniae, Citrobacter freundii, letter

**To the Editor:** The activity of carbapenem has been compromised because of the emergence of carbapenemases (*1*). Since 1995, carbapenem resistance has been identified among 77 *Klebsiella pneumoniae* isolates and 1 *Citrobacter* *freundii* clinical isolate in Argentina (WHONET-Argentina Network). However, until now, none had produced a carbapenemase.

*K. pneumoniae* carbapenemase-1 (KPC-1) was first detected in a *K. pneumoniae* strain isolated in North Carolina in 2001 (*1*). Since that time, several reports of KPCs worldwide have been made, including in South America (*1*). We report on KPC-2–producing *K. pneumoniae* and *C. freundii* clinical isolates in Argentina.

A 36-year-old woman with systemic lupus erythematosus and chronic renal failure was admitted to the Sanatorio Mitre in Buenos Aires in September 2006 for a kidney transplant. Two months after the transplant, intraabdominal collection obtained during a surgical procedure yielded a carbapenem-susceptible *Escherichia coli* isolate, after which meropenem therapy was initiated (1 g/day). After 16 days of treatment, an infection developed at the patient’s surgical site (per US Centers for Disease Control and Prevention criteria, available from www.cdc.gov/ncidod/dhqp/pdf/guidelines/SSI.pdf). *C. freundii* M9169 and *K. pneumoniae* M9171 were both isolated from the same specimen obtained from the surgical site. Because carbapenemase production was suspected, carbapenem treatment was stopped, and the infection was treated with local antiseptic and drainage for 20 days; the patient was discharged from the hospital in January 2007. Neither the patient nor her relatives or hospital staff had been in the United States before the emergence of these strains.

By using disk diffusion (*2*) (Mueller-Hinton agar and disks obtained from Difco and BBL, respectively; Becton, Dickinson and Co., Franklin Lakes, NJ, USA), we determined that *K. pneumoniae* M9171 was resistant to all antimicrobial drugs except amikacin, tetracycline (*3*), and tigecycline (US Federal Drug Administration criteria, susceptible >19 mm ). *C. freundii* M9169 remained susceptible to carbapenems, cefepime, ciprofloxacin, aminoglycosides, chloramphenicol, tetracyclines, and tigecycline but displayed resistance to ceftazidime (CAZ), cefotaxime (CTX), nalidixic acid, and trimethoprim-sulfamethoxazole. When tested with an AmpC-type β-lactamase inhibitor (*4*), both strains showed synergism between 3-aminophenylboronic acid (APB, Sigma-Aldrich, St. Louis, MO, USA) disks and CTX, CAZ, and carbapenems when placed 20 mm apart (center to center). The same synergism was observed for *K. pneumoniae* D5/07, a reference KPC-2–producing strain (College of American Pathologists Quality Control Assurance Program), but not among *E. coli* ATCC 25922. A CMY-2–producing *K. pneumoniae* C2 control strain (*5*) displayed APB synergism against only CTX and CAZ, not carbapenems.

The MICs of carbapenems (*6*) (agar dilution), confirmed disk diffusion results showing a >3 doubling-dilution decrease after the addition of APB (300 μg/mL) for *K. pneumoniae* M9171, *C. freundii* M9169, and *K. pneumoniae* D5/07, but not for *K. pneumoniae* C2 and *E. coli* ATCC 25922. Clavulanate (4 μg/mL) reduced only meropenem and ertapenem, and imipenem MICs of *C. freundii* M9169 and *K. pneumoniae* D5/07, respectively (Table).

**Table:** Antibiotic susceptibility, isolectric focusing of β-lactamases and PCR of antimicrobial resistance determinants in *Klebsiella pneumoniae*, *Citrobacter freundii* clinical isolates, *Salmonella* transconjugants, and recipient and control strains*

Type of testing	Clinical isolates			Transconjugants and recipient strains					Control strains		
	*K.p.* M9171	*C.f.* M9169		*Salmonella* M9204†	*Salmonella* M9190‡		*Salmonella* M1744		*K.p.* D5/07§	*K.p.* C2¶	*E. coli* ATCC 25922
	MICs (µg/mL)										
Antimicrobial agent											
Imipenem	32	1.0		1.0	1.0		0.12		2.0	0.12	0.25
Impenem/clavulanate	32	0.25		0.25	0.25		0.12		0.5	0.12	0.25
Impenem/APB	2.0	0.12		0.25	0.12		0.12		0.12	0.12	0.25
Imipenem/EDTA#	32	0.25		1.0	1.0		0.12		2.0	0.12	0.12
Meropenem	32	1.0		0.5	1.0		0.015		2.0	0.03	0.03
Meropenem/clavulanate	32	0.12		0.03	0.06		0.015		1.0	0.03	0.03
Meropenem/APB	1.0	0.06		0.03	0.03		0.015		0.12	0.03	0.03
Meropenem/EDTA#	32	1.0		0.5	1.0		0.015		2.0	0.03	0.03
Ertapenem	128	2.0		1.0	1.0		0.008		16	0.06	0.015
Ertapenem/clavulanate	128	0.25		0.03	0.06		0.008		16	0.06	0.008
Ertapenem/APB	8.0	0.008		0.015	0.03		0.008		0.5	0.03	0.008
Cefoxitin	64	64		4.0	8.0		2.0		ND	ND	4
Ceftazidime	256	16		16	32		0.06		ND	ND	0.25
Ceftazidime/clavulante	16	32		0.5	0.5		0.06		ND	ND	ND
Cefepime	32	4.0		16	16		0.03		ND	ND	0.03
Cefepime/clavulanate	16	0.25		0.5	0.25		0.03		ND	ND	ND
Tigecycline**	1.0	0.25		ND	ND		ND		ND	ND	0.25
	Isolectric focusing results										
pI band††	5.4 + 6.7 + 7.6	5.4 + 6.7 + >9.0		6.7		6.7	None		ND	ND	None
	PCR results										
Β-lactamase											
*bla*_KPC_	+	+		+		+	–		+	–	ND
*bla*_ampC_	–	+ CIT/ CMY		–		–	–		–	+ CIT/ CMY	ND
*bla*_PER-2_	+	–		–		–	–		ND	ND	ND
*bla*_SHV_	+	–		–		–	–		ND	ND	ND
*bla*_TEM-1_	+	+		–		–	–		ND	ND	ND

*K.p., *Klebsiella pneumoniae*; *C.f.*, *Citrobacter freundii*; *E. coli*, *Escherichia coli*; APB, 3-aminophenylboronic acid; IEF, isoelectric focusing; pI, isoelectric point; ND, not determined; +, positive; –, negative. †M9204 transconjugant derived from *K. pneumoniae* M9171. ‡M9190 transconjugant derived from *C. freundii* M9169. §Control strain producing KPC-2. ¶Control strain producing CMY-2. #EDTA 0.4 mmol/L. **Microdilution (JustOne, Trek Diagnostic Systems, Cleveland, OH, USA). Breakpoints according to US Food and Drug Administration (susceptible <2 μg/mL). ††Underlined pI bands indicated activity against third-generation cephalosporins

Isoelectric focusing (IEF) showed that both isolates produced several β-lactamases (Table), including a common enzyme with ESBL activity (isoelectric point [pI] 6.7 by a substrate-based iodometric method) (*7*). *K. pneumoniae* M9171 coproduced another ESBL band at pI 5.4 (*7*).

Beta-lactamases were characterized by PCR specific for KPC (forward primer 5′-AACAAGGAATATCGTTGATG-3′; reverse primer 5′-AGATGATTTTCAGAGCCTTA-3′), PER-2, SHV, and TEM (*7*). Both strains were PCR-positive for KPC and TEM. PER-2 and SHV were amplified in *K. pneumoniae* M9171. Because of the APB inhibition observed, strains were tested for plasmid-mediated AmpC genes (*8*). The amplicon for the CIT/CMY primers was observed for *C. freundii* M9169 (expectable cross-amplification with chromosomal AmpC) (Table). KPC-type PCR product (916 bp) obtained from *K. pneumoniae* M9171 was sequenced and identified as KPC-2 (*1*).

A wild-type *Salmonella* clinical isolate (M1744) was chosen for conjugational purpose because it naturally lacks AmpCs . Conjugation resulted in the transfer to M1744 of penicillins and third-generation cephalosporin resistance from both clinical isolates (frequency 10^–4^ to 10^–5^, when selected with ampicillin [50 μg/mL] in *Salmonella–Shigella* medium). Transconjugants showed the acquisition of an ≈70-kb plasmid, which was present in both clinical isolates (*9*). Transconjugants displayed an APB double-disk augmentation trait, further observed by MIC. Only the KPC enzyme was transferred (unique band at pI 6.7) by IEF, which was confirmed by PCR. The absence of plasmid-mediated AmpC genes was confirmed by PCR (Table). No other resistances to non–β-lactam agents were cotransferred.

Carbapenemase activity of crude extracts was measured at 30°C by following 0.4 mmol/L imipenem or ertapenem hydrolysis at 300 nm in 10 mmol/L HEPES (pH 7.5). Addition of 4 mmol/L APB resulted in inhibition of carbapenemase activity of *K. pneumoniae* M9171, *C. freundii* M9169, both transconjugants, and *K. pneumoniae* D5/07, but not a VIM-11 control run in parallel (*10*).

This study identified KPC β-lactamase, which was possessed by 2 strains recovered from 1 patient, in Argentina. Detection of this carbapenemase could become cumbersome because carriage of these genes does not always confer obvious resistance. Moreover, an unusual phenotype was observed in this study; boronic inhibition was associated with the sole presence of KPC. Microbiologists should be aware of cross-reactions (synergism) between APB and KPC that could lead to the false assumption of AmpC-type β-lactamase production, thereby underestimating the presence of this carbapenemase.
